# Editorial: Exploring novel in vitro models for cystic fibrosis research

**DOI:** 10.3389/fphar.2026.1779926

**Published:** 2026-02-20

**Authors:** Yuncheng Man, Domenico Mattoscio, Roberto Plebani

**Affiliations:** 1 Wyss Institute for Biologically Inspired Engineering at Harvard University, Boston, MA, United States; 2 Vascular Biology Program and Department of Surgery, Boston Children’s Hospital and Harvard Medical School, Boston, MA, United States; 3 Department of Medical, Oral and Biotechnological Sciences, Center for Advanced Studies and Technology, University “G. d’Annunzio” Chieti-Pescara, Chieti, Italy

**Keywords:** CFTR, cystic fibrosis, human nasal epithelial cell, *in vitro*, modulators, N1303K CFTR, oxidative stress, solid lipid nanoparticle

The introduction of highly effective CFTR modulator therapies has significantly transformed the management of Cystic Fibrosis (CF). However, these treatments may lead to different clinical results, depending on factors including the specific CFTR mutations, tissue types involved, and the individual response of patients. To overcome these challenges and facilitate the translation of next-generation CF therapeutics into the clinics, investigators are moving towards more complex *in vitro* systems. This topical series, “Exploring Novel *In Vitro* Models for Cystic Fibrosis Research”, presents a compilation of research articles reflecting substantial scientific progresses in addressing these challenges. The scope extends beyond conventional drug testing and disease modeling, to address critical aspects such as model robustness, novel drug delivery systems, and standardized metrics for evaluating therapeutic outcomes. These are all foundational elements for advancing towards next-generation microphysiological platforms in preclinical research.

A central challenge in CF treatment is understanding why a specific modulator therapy is effective in one organ system but less effective in another. Pranke et al. provide pivotal insights into this issue by investigating the N1303K CFTR mutation. Although the triple-combination therapy Elexacaftor/Tezacaftor/Ivacaftor (ETI) enhances lung function, its impact on sweat chloride levels is modest. Using novel, airway- and sweat gland-derived cell lines, the work demonstrates a clear tissue-specific response to ETI. Airway-based models showed significantly improved chloride transport, consistent with clinical improvements in lung function. Conversely, in sweat gland models, ETI failed to rectify the folding defect associated with N1303K-CFTR. This critical discovery underscores the importance of employing diverse physiologically relevant *in vitro* models to decipher the complexities of therapeutic mechanisms, reinforcing the limitations of universal approaches to CF modeling and treatment. Moreover, this finding in a controlled 2D system warrants further investigation using more sophisticated, next-generation models, such as organ-on-a-chip with different tissue origins. Such an approach could provide molecular insight into the potential role of tissue-specific elements, including the extracellular matrix or immune cells, in differing drug responses. This could also pave the way for more comprehensive systemic therapies.

In addition to a better understanding of drug mechanisms, enhancements in delivery systems will play a crucial role in the progression of CF pharmacology. Parhiar et al. propose an innovative approach with their Solid Lipid Nanoparticle (SLN) formulation of Ivacaftor that aims to address challenges associated with the drug’s solubility and delivery in CF contexts. Their work demonstrated that this innovative formulation possesses high structural stability, good drug encapsulation efficiency, and sustained-release performance, which may enhance the bioavailability of drugs. Overall, this work highlights that next-generation delivery systems are integral complements to sophisticated *in vitro* modeling, together enabling more effective drug evaluation and accelerating clinical translation. Although initial characterization is typically conducted using simplified models, the true effectiveness of the delivery system, encompassing muco-penetration, epithelial cell uptake, and sustained release, can only be comprehensively evaluated in a physiologically relevant and personalized 3D model. This will facilitate more predictive drug testing and enhance the translational relevance of preclinical findings.

To further promote the clinical implementation of these sophisticated, patient-specific platforms, it is imperative to first characterize the key components of their cellular structure. Human Nasal Epithelial (HNE) cells are non-invasive surrogates for bronchial epithelial cells, but their use is limited due to insufficient validation in comparison with other more established methodologies. Barillaro et al. address this gap through a comparative study of paired nasal and bronchial samples derived from individual patients. Their findings demonstrate that, although HNE cells exhibit reduced CFTR functionality compared to bronchial cells, there is a strong correlation in the magnitude of the response to modulators. These critical insights support the use of HNE cells in preclinical drug testing, while also emphasizing the importance of consistency in experiment design, since even small changes in the experimental conditions under which cells are grown or the procedures applied can considerably influence the outcomes. In the context of *in vitro* CF research models, the establishment of rigorous criteria for experimental design and validation of primary cells assumes particular significance. This is largely due to the necessity of ensuring the reproducibility and clinical significance of results, a concern that is further amplified when more complex 3D cultures from patient-derived cells are employed.

Similarly, standardized and validated methods for measuring oxidative stress, a CF hallmark, are needed. In a timely review, Rubin et al. detail a broad range of analytical techniques used to quantify reactive oxygen species, oxidative injury, and antioxidant defenses in CF. The authors reviewed the advantages and limitations of different assays and models and discussed how inconsistencies in experiments may arise. As the field evolves towards the use of multi-parametric readouts in organoid and organ-on-a-chip platforms, the development of validated, reproducible measures of oxidative stress will be of critical importance to ensure the ability of advanced models to provide clinically relevant information.

In summary, the Research Topic of articles in this Research Topic underscores the rapid evolving role of advanced *in vitro* models in the next era of CF research. The transition from conventional 2D cultures to advanced 3D microphysiological systems, such as organoid and organ-on-a-chip is a remarkable progress in the development of more predictive and personalized CF models. These advances have the potential to establish a connection between molecular findings and individual therapeutic approaches, thereby accelerating the implementation of personalized CF therapies.

